# Benchmarking the acceleration of materials discovery by sequential learning†

**DOI:** 10.1039/c9sc05999g

**Published:** 2020-01-29

**Authors:** Brian Rohr, Helge S. Stein, Dan Guevarra, Yu Wang, Joel A. Haber, Muratahan Aykol, Santosh K. Suram, John M. Gregoire

**Affiliations:** Accelerated Materials Design and Discovery, Toyota Research Institute Los Altos CA USA santosh.suram@tri.global; Joint Center for Artificial Photosynthesis, California Institute of Technology Pasadena CA USA gregoire@caltech.edu; Division of Engineering and Applied Science, California Institute of Technology Pasadena CA USA

## Abstract

Sequential learning (SL) strategies, *i.e.* iteratively updating a machine learning model to guide experiments, have been proposed to significantly accelerate materials discovery and research. Applications on computational datasets and a handful of optimization experiments have demonstrated the promise of SL, motivating a quantitative evaluation of its ability to accelerate materials discovery, specifically in the case of physical experiments. The benchmarking effort in the present work quantifies the performance of SL algorithms with respect to a breadth of research goals: discovery of any “good” material, discovery of all “good” materials, and discovery of a model that accurately predicts the performance of new materials. To benchmark the effectiveness of different machine learning models against these goals, we use datasets in which the performance of all materials in the search space is known from high-throughput synthesis and electrochemistry experiments. Each dataset contains all pseudo-quaternary metal oxide combinations from a set of six elements (chemical space), the performance metric chosen is the electrocatalytic activity (overpotential) for the oxygen evolution reaction (OER). A diverse set of SL schemes is tested on four chemical spaces, each containing 2121 catalysts. The presented work suggests that research can be accelerated by up to a factor of 20 compared to random acquisition in specific scenarios. The results also show that certain choices of SL models are ill-suited for a given research goal resulting in substantial deceleration compared to random acquisition methods. The results provide quantitative guidance on how to tune an SL strategy for a given research goal and demonstrate the need for a new generation of materials-aware SL algorithms to further accelerate materials discovery.

## Abstract 2

Benchmarking metrics for materials discovery *via* sequential learning are presented, to assess the efficacy of existing algorithms and to be scientific in our assessment of accelerated science.

## Graphical abstract

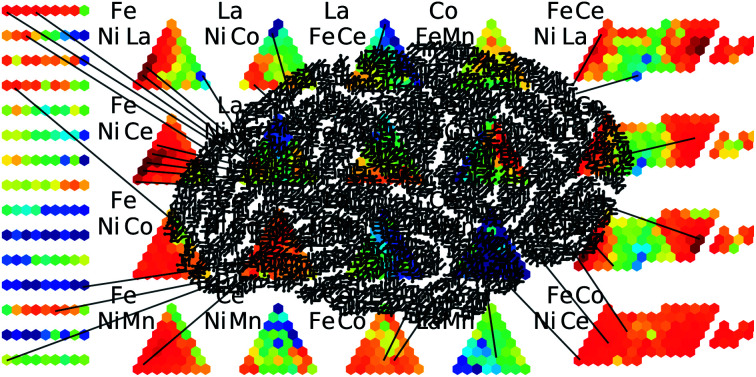

## Introduction

Accelerating materials discovery is of utmost importance for realization of several emergent technologies, particularly to combat climate change through the adoption of zero or negative emission technologies such as hydrogen driven cars and other means of clean chemical energy generation, storage and utilization. One method of accelerating materials research is through integration of automated experiments^1–4^ that are guided by artificial intelligence (AI).^5,6^ Specifically, AI sampling strategies^7,8^ hold great promise for resource-constrained activities such as materials research due to their potential to minimize the number of experiments necessary for achieving a desired objective.^9^ Sequential learning (SL) methods wherein a machine learning model guides experiment at each iteration based on pre-sampled data is a promising approach to accelerate materials research. SL has been used to reduce the number of expensive density functional theory (DFT) calculations needed to find materials with desired bulk and interfacial properties,^10–12^ to fit potential energy surfaces^13^ as well as to compute an improved force field for small molecules.^14^ Recent demonstrations in chemistry include optimization of the drug-likeness and synthesizability of small molecules,^15^ and of the efficiency of organic light emitting diode molecules.^16^ SL methods^7,8^ have also been paired with physical experiments to improve the efficiency of materials discovery as demonstrated by a factor of 2 to 5 reduction in the number of experiments required to discover efficient thermoelectric materials, superconductors, and steels with high fatigue strength,^17^ and to discover new Pb-free BaTiO_3_ (BTO) based piezoelectrics with large electrostrains.^18^ SL has even been paired with robotic experiments to create a fully autonomous organic reaction searching system for exploring chemical reactivity and synthesis of new molecules,^19^ and optimization of organic photovoltaics.^20^ Recent reviews^7,8^ highlight the breadth of demonstrated applications of SL in the chemical sciences, though there have been relatively limited demonstrations in solid state materials science, where the autonomous optimization of carbon nanotube growth is a seminal example.^21,22^ SL paired with robotic experiment has the potential to greatly accelerate experimental discovery of new materials and chemicals,^23^ which points to the importance of benchmarking these methods and understanding their behavior in different materials research settings.

In SL, a model (or an ensemble of models) is updated iteratively to achieve an objective ranging from performance optimization to development of an accurate prediction model. The update could be *via* an incremental update rule or retraining of the model with the incrementally expanded training data.^7^ The incremental update rules used in the former setting restricts the ML models to Bayesian based approaches. In addition, incremental update rules typically focus on improving overall prediction of the model, making this setting less flexible for various research objectives. Thus, the latter, more flexible setting is more common in materials research implementations of SL, and is a convenient choice for exploring various research objectives and ML models. We adopt sequential learning in the latter setting to enable comparison of metrics across a variety of research objectives and machine learning (ML) algorithms.

In the present work we benchmark SL for oxygen evolution reaction (OER) catalyst discovery in high-dimensional composition spaces,^24^ where we define discovery of a good catalyst to be identification of a composition whose activity is in the top percentile of all compositions in the search space. The comprehensive datasets that enable simulation and benchmarking of SL originate from previously published high throughput experimentation techniques.^24^ Initial benchmarking studies involve comparison of three complementary ML regression models: random forest (RF),^25^ Gaussian process (GP),^25^ and query by committee using linear ensemble (LE) methods. Qualitatively similar behavior among the 3 models, and the excellent performance and computational efficiency of the RF model motivated its selection for further benchmarking studies on different exploration *vs.* exploitation settings, as well as evaluation of three additional catalyst datasets to investigate the generality of the benchmarking results with respect to composition search space. Efficiency gains with SL for the various research tasks vary significantly, from approximately 20-fold acceleration to drastic deceleration in the number of catalyst measurements required to reach a specific goal, with sensitivity to random initialization, indicating where scientists need to tread carefully in the incorporation of SL. The use of custom algorithms such as PHOENICS for chemistry experiments^26^ is an excellent example of developing algorithms tailored to a specific application, which can lead to improved performance and model stability. Establishing chemically meaningful representations of the search space and improving uncertainty quantification also emerge as key research areas to facilitate more pronounced acceleration of materials research.^27^

## Experimental and computational

### Synthesis and catalyst experiments

**Fig. 1:**
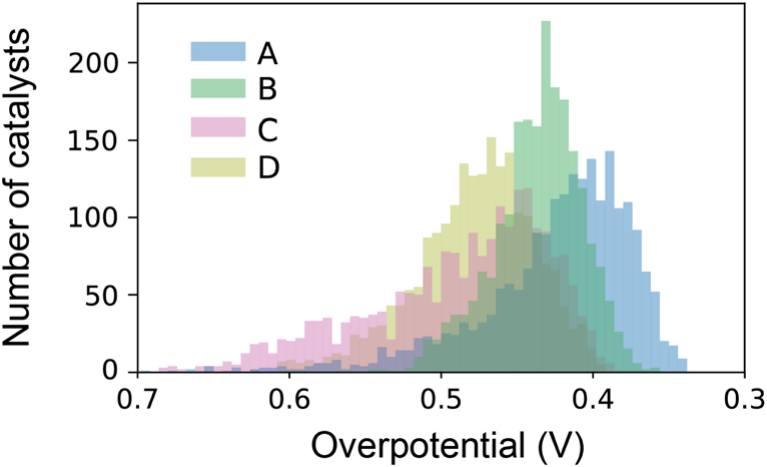
Distribution of catalyst activities over the four datasets used for benchmarking of sequential learning algorithms.

The datasets for simulated SL were constructed from high throughput experiments described previously.^24,28^ Parallel synthesis and processing of a composition library proceeded with inkjet printing^29^ of elemental precursors to produce a discrete library with 2121 unique compositions comprising all possible unary, binary, ternary and quaternary compositions from a 6 element set with 10 at% intervals. Following conversion to metal oxide samples *via* calcination at 400 °C for 10 hours, accelerated aging of the catalysts is performed *via* parallel operation for 2 hours.^30^ Subsequent serial characterization using a scanning droplet cell provides the OER overpotential at 3 mA cm^−2^ (per the geometric area of 1 mm^2^ catalyst samples) in pH 13 electrolyte (0.1 M sodium hydroxide + 0.25 M sodium sulfate), the negative of which provides the figure of merit (FOM) for each composition with increasing FOM value corresponding to better catalytic activity. Each collection of 2121 FOMs is treated as an independent dataset for sequential learning simulation, and each such dataset contains considerable catalyst composition diversity with 6, 15, 20, and 15 catalyst composition spaces containing 1, 2, 3 and 4 cation elements, respectively. The different datasets, their respective identifier in our database,^28^ and the 6-element composition system are shown in Table 1. The distribution of catalyst overpotentials in each dataset is shown in Fig. 1.

**Table 1:** Mapping of labels to composition spaces and plate ID. The compositional complexity of the best 21 catalysts in each dataset is noted by number of these catalysts with 1, 2, 3 and 4 cation elements in their compositions

Plate ID	Label	Composition system	Num. of top 1% with *N* cations			
			*N* = 1	*N* = 2	*N* = 3	*N* = 4
3496	A	Mn–Fe–Co–Ni–La–Ce	0	0	8	13
3851	B	Mn–Fe–Co–Ni–Cu–Ta	0	3	13	5
3860	C	Mn–Fe–Co–Cu–Sn–Ta	0	1	6	14
4098	D	Ca–Mn–Co–Ni–Sn–Sb	0	0	2	19

**Fig. 2:**
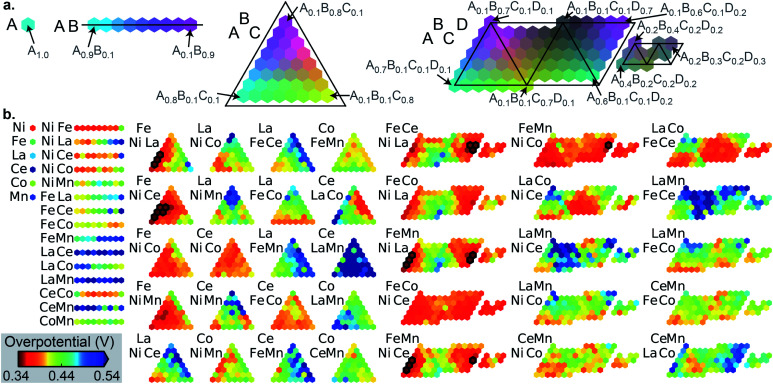
(a) Illustration of the composition plotting schema for compositions containing 1, 2, 3 and 4 cation elements, with example compositions noted for the placeholder element names A, B, C and D. (b) The 2121 OER overpotentials in dataset A for the 6, 15, 20 and 15 compositions spaces containing 1, 2, 3 and 4 cations, respectively. The 21 compositions outlined in black comprise the top percentile in catalyst activity. The overpotential color scale is noted at bottom left.

The number of the 21 top catalysts for each compositional complexity, *i.e.* 1, 2, 3 or 4 cations, is noted in Table 1 for each dataset. While plates B and C have some representation of 2-cation catalysts in the top percentile, the 3 and 4-cation catalysts contain most of the top catalysts, illustrating the utility of compositional complexity in catalyst optimization. To visualize the compositional search space and illustrate the existence of top catalysts in various composition regions, the overpotentials of the 2121 compositions in dataset A are shown in Fig. 2. The top 21 catalysts (lowest 1 percentile in overpotential) are indicated, revealing the existence of top catalysts in 6 distinct composition spaces, which illustrates the importance of identifying local maxima in a search for all top catalysts.

While the ensuing benchmarking of SL ignores the chemistry of the catalysts, with each catalyst represented by its unlabeled composition, we briefly comment here on the chemistry underlying the composition–activity trends. Ni and Fe oxides are well known to form mixed oxyhydroxides in alkaline electrolytes that are effective OER catalysts.^31^ In Fig. 2, the 6 compositions spaces with top catalysts all contain Ni and Fe, and while Ni–Fe compositions exhibit appreciable activity, the results illustrate that incorporation of a variety of other elements and combinations thereof can further improve activity. La^32^ and Ce^33^ are the most commonly occurring additional elements in the top catalysts, and the mechanism for increased activity is likely related to the modification of the redox potential of active transition metal oxyhydroxide active sites, as recently illustrated with *in situ* and *operando* spectroscopies.^34^ Dataset C (see Fig. S2†) exhibits more complex variation in activity with different element combinations. Mn–Cu and Mn–Co–Cu composition spaces contain the best catalysts with 2 and 3 cations, respectively, all with Mn-rich compositions. Yet the Mn-free composition space Fe–Co–Cu–Sn exhibits high activity in nearly all of its compositions. The composition–activity relationships in these datasets are difficult to interpret without additional structural characterization, which is substantially more expensive to acquire than the catalyst activities noted here. A broader vision of the present work is to accelerate the learning of composition–activity relationships to guide design of structure characterization experiments, as discussed further below.

### Sequential learning models

The SL framework is designed to enable facile variation in both the machine learning model and acquisition function and is implemented under the assumptions of a discretized search space that represents all possible experiments, which we refer to as the sample set of size *N*. Each sample from this set is represented by its experiment coordinate, which is the 6 dimensional composition vector. We consider a scalar figure of merit (FOM) for each sample and note that the framework can be extended to multi-objective optimization.^26^ We also limit the present benchmarking effort to single-selection learning commensurate with serial experimentation. While multi-sample or “batch” selection strategies may also be benchmarked by this approach, the lack of a general cost function for experiment parallelization limits quantification of the relative acceleration provided by batched active learning.^5,6^

Each SL algorithm is implemented into the framework by assuming no pre-existing FOM measurements beyond random selection of two initial experiments (*i* = 0, 1). Subsequent experiment selection proceeds through iteration of a 4-step procedure:

(1) Measurement of the FOM for the selected experiment

(2) Training of ML model with updated dataset

(3) Evaluation of ML model at all non-sampled coordinates (*j*) to obtain each predicted FOM value, *μ*_*j*_, and its uncertainty *σ*_*j*_

(4) Selection of the next experiment *via* the acquisition function, which identifies the coordinate *j* that maximizes the quantity in the upper confidence bound setting: *λμ*_*j*_ + (1 − *λ*)*σ*_*j*_ where *λ* is a hyperparameter that can be varied from 0 to 1 to tune the exploration-exploitation tradeoff. The SL cycle *i* results in the measurement of the FOM for a newly-selected point in the search space, thereby increasing the size of the training set to *i* + 1 samples.

This SL technique can be implemented with any machine learning model that provides a predicted FOM value and uncertainty of that prediction for each input coordinate. Here we choose three models that cover a breadth of algorithmic approaches to SL: a query-by-committee type linear ensemble method (LE), Gaussian process as a representative Bayesian method (GP), and a random forest model (RF). Briefly, our LE implementation consists of an ensemble of *k* = 40 linear regressors of the form 
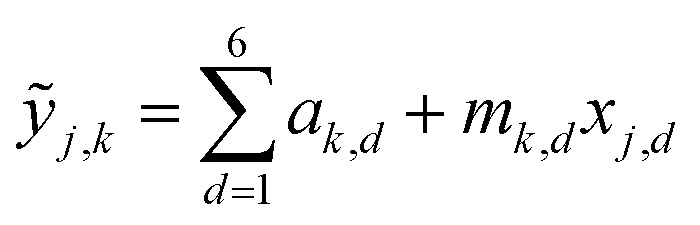
 for predicting the FOM based on the *j*^th^ input vector *x*_*j*,*d*_ where *d* denotes the composition dimension of the input vector. Each regressor is only fit using a random selection of 70% of the previously sampled data, and the committee of *k* linear regressors is evaluated at each coordinate, creating a collection of values whose mean and standard deviation are taken as *μ*_*j*_ and *σ*_*j*_, respectively. The RF model in the present work uses the scikit-learn^25^ (version 0.21.2) implementation with 50 estimators and the default values for all other parameters. Similar to the LE model, the mean and standard deviation of the individual decision trees' predictions provide *μ*_*j*_ and *σ*_*j*_, respectively. The Bayesian GP model directly provides *μ*_*j*_ and *σ*_*j*_. The scikit-learn^25^ implementation of the Gaussian process regressor is used with a constant combined with a Matern 5/3 kernel with interaction length of 1. The noise parameter, alpha, was set to 0.01.

### Performance metrics for active learning

To quantify the performance of the SL algorithms for different research objectives, we introduce four complementary active learning metrics (ALMs) that can be evaluated at each SL cycle and can be applied more broadly to evaluate different active learning methods. The decision efficiency metric, ^*de*^ALM, is based on a desire to perform the experiment that will provide the highest FOM among the non-sampled materials. At cycle *i*, the chosen sample will have FOM greater than or equal to a fraction *f*_*i*_ of the *N* − *i* available samples, and defining ^*de*^ALM_*i*_ = 2 × *f*_*i*_ − 1 provides a (−1, 1) scale where 1 is optimal and 0 is the expected value for random selection.

The second metric, ^*any*^ALM, is based on the discovery of any catalyst from the top 1 percentile of the FOM from the full dataset, where we use the 1% threshold as a nominal definition of a good catalyst. For a given SL run, the first of these good catalysts is found at cycle *i*, and to provide a continuous metric for a given SL algorithm, ^*any*^ALM_*i*_ is calculated as the fraction of randomly initialized runs that have measured any catalyst from the top percentile by cycle *i*.

The third metric, ^*all*^ALM, similarly considers the top percentile and is based on identifying all such catalysts. For a given SL run ^*all*^ALM_*i*_ is calculated as the fraction of the top percentile catalysts that have been measured by cycle *i*.

The fourth metric, ^*model*^ALM, is based on developing an accurate predictive model for all samples yet to be measured. Since ^*any*^ALM and ^*all*^ALM are between 0 and 1 (inclusive), ^*model*^ALM is calculated from an error function *E* as *E*_full_/*E*_*i*_, the ratio of the model error when training with the full dataset and with the sequential learning dataset up to cycle *i*. For the present work, the error function is taken to be the mean absolute error (MAE) over non-measured samples, and the scaling factor *E*_full_ is calculated using a random 70% train and 30% test set, where the MAE is calculated on the test set. By definition random sampling of 70% will on average produce a ^*model*^ALM of 1. To ensure sufficient test set size for the error function calculation, this metric is not evaluated from SL cycles beyond 70% of the dataset. While this metric can be deployed with nominally any ML model, the scaling factor *E*_full_ depends on the ML model, prompting our use of the MAE (*E*_*i*_) when comparing different ML models and ^*model*^ALM when using a fixed ML model (RF in this case) and varying other aspects such as acquisition function and datasets.

While ^*de*^ALM has a comparison to random selection built into its definition, for the other three metrics each SL algorithm is compared to a baseline model of random sample selection using two complementary metrics for measuring the factor by which SL improves performance. Taking 
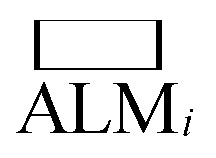
 to be the ALM values for random sampling, the ratio 
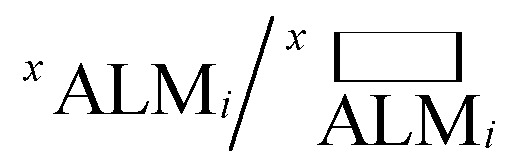
 is referred to the enhancement factor (^*x*^EF_*i*_) for metric *x*, which quantifies the improvement when operating experiments at a given budget for the number of experiments. The complementary mode of operation is to specify a required value for ALM and consider the number of experiments needed to meet the requirement. The cycle ratio *r*/*i* is the acceleration factor (^*x*^AF_*y*_) in terms of the number of required cycles for attaining a particular value ^*x*^ALM, *i.e.* where ^*x*^ALM_*i*_ = *y* and 
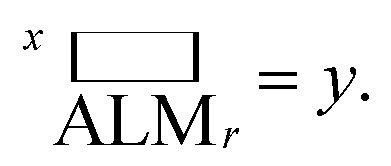
 The random baseline is calculated as described in the ESI† for *x* = *any* and *all* and simulated for *x* = *model*.

### Simulations and aggregation

As described above, ^*any*^ALM is best defined as an average over random initializations, motivating us to use an aggregation scheme for ^*any*^ALM, ^*all*^ALM, and ^*model*^ALM wherein each SL algorithm is run 50 times with independent, random initializations. Averaging ^*x*^ALM over sets of 10 runs enables ^*any*^ALM and ^*all*^ALM to be specified in intervals of 0.1 and 0.005, respectively, and 50 such sets (10 random 5-fold splits from the 50 runs) are used to characterize the variation in each ^*x*^ALM. This variation is visualized by plotting the median value as well as shaded regions representing the 6^th^ to 94^th^ percentile, *i.e.* removing the top 2 and bottom 2 values from each set of 50 values for ^*x*^ALM_*i*_.

## Results

**Fig. 3:**
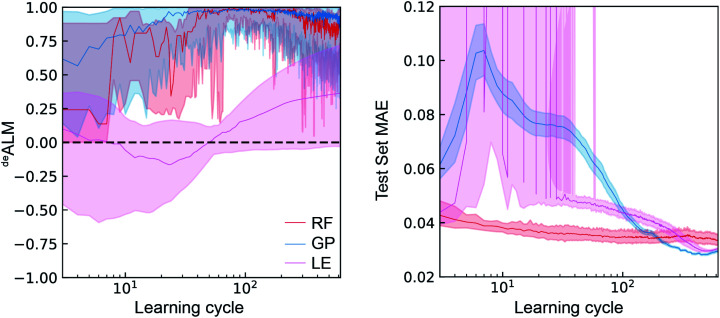
The active learning metrics ^*de*^ALM and test set MAE (V) for 700 measurement cycles are shown for each of the three ML models using dataset A. The median value (solid line) as well as the 6^th^ to 94^th^ percentile (shaded region) are based on 50 random initializations of each sequential learning model.

In the setting of catalyst discovery, we begin with comparison of ^*de*^ALM of different ML algorithms obtained from simulated SL runs using dataset A. The ^*de*^ALM is intended to evaluate the decision making under a mode of research where the goal is to identify the best possible material in each experiment cycle, and the comparison of the three ML models with *λ* = 0.5 is shown in Fig. 3. The median value over various random initializations is shown along with the variability at each learning cycle. The ^*de*^ALM of each model is highly variable when only a small number of learning cycles have been carried out. The RF and GP models start to provide consistent selection of samples from the top quartile of available catalysts (^*de*^ALM > 0.5) after about 40 learning cycles, whereas LE needs nearly 300 cycles to reach the same performance.

With regard to the test set MAE metric, when only a small number of active learning cycles have been carried out, the RF model performs the best and displays little variation due to random initialization. It outperforms the GP and LE models until 200 and 300 learning cycles, respectively. The LE model is unable to make reasonable predictions until about 40 learning cycles. After about 200 learning cycles, the GP model gives the lowest test MAE as well as the smallest variation due to random initialization. Despite being a simpler model, the LE model outperforms the RF model after about 300 cycles with respect to test set MAE, perhaps due to only 70% of the previously sampled data being used for training each linear regressor. Comparison of the MAE with ^*de*^ALM shows a general trade-off between choosing the best catalysts and improving test set MAE. The ^*de*^ALM improves with number of cycles for RF and GP, reaching a value of above 0.75 consistently at 100 cycles. The ^*de*^ALM then decreases possibly because the algorithm has to choose amongst equally bad catalysts.

In the case of LE, the ^*de*^ALM does not improve until 40 cycles, possibly because of the minimum number of data points necessary for an invertible solution for linear regression. The ^*de*^ALM improves after 40 cycles but the ^*de*^ALM is not consistently greater than 0.75 even after 500 cycles. Avoiding composition regions with poor activity hampers improvement of model prediction, as demonstrated by relatively little improvement in MAE with increased number of cycles. Between cycles 40 and 200, the GP model exhibits the most substantial deviation from this trade-off by substantially improving MAE while maintaining a high ^*de*^ALM, likely due to better uncertainty quantification in this Bayesian model as compared to the ensemble-based uncertainty calculation of the RF and LE models.

**Fig. 4:**
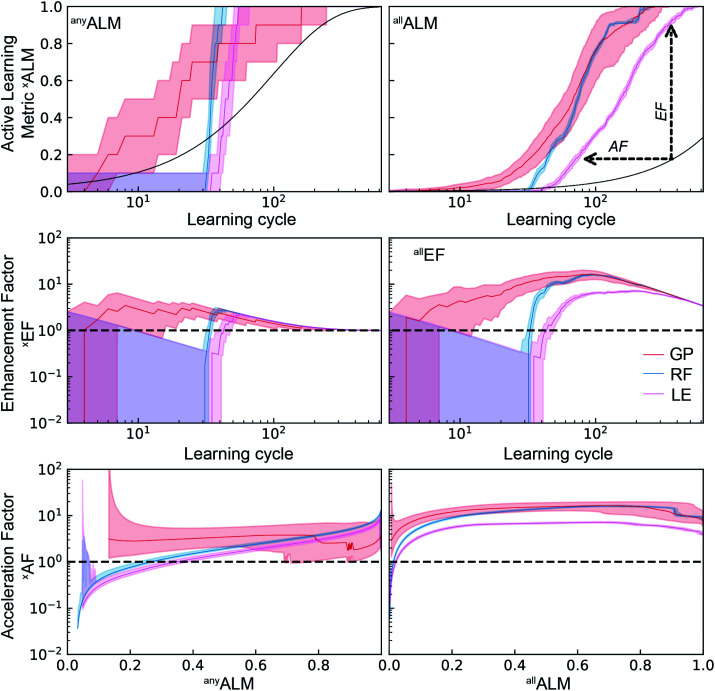
Top) The active learning metrics ^*any*^ALM and ^*any*^ALM are shown for each of 3 ML models using dataset A. The median value (solid colored line) as well as the 6^th^ to 94^th^ percentile (shaded region) are based on 50 random initializations of each sequential learning model. The solid black line corresponds to the expectation value from random sampling (see ESI†). (Middle) The black dashed line shows the expectation value of each ALM for random sampling. The enhancement factors (Middle) and acceleration factors (Bottom) with respect to random sampling.

As a performance metric, ^*de*^ALM evaluates the ability to identify top catalysts but is not amenable to quantitative analyses of the factor by which SL improves research. For this analysis we consider the metrics ^*any*^ALM and ^*all*^ALM calculated from the same SL runs shown in Fig. 3. The results shown in Fig. 4 show qualitatively similar performance for the three ML models. Finding any catalyst from the top percentile is a relatively easy task and shortly after a ML models gains some predictive ability, it is highly likely to identify a top catalyst, with GP, RF, and LE ML models all having median ^*any*^ALM exceeding 80% by 35 to 55 learning cycles. Finding all of the top catalysts is a more challenging task where the relatively advanced algorithms RF and GP excel compared to LE, typically finding 80% of the top catalysts by approximately 100 cycles for RF and GP, as opposed to over 200 cycles for LE. These benchmarks for GP and LE are quite insensitive to random initialization while the number of required cycles for RF varies by 10 s of cycles with different initializations.

The signals corresponding to expectation values of each ALM from random sample selection (see ESI†) are shown in dashed black lines, enabling comparisons to random selection including the enhancement and acceleration factors, as shown in Fig. 4. These complementary modes of comparison are based on experimentation with a fixed experiment budget and with a fixed research objective using a variable experiment budget, respectively. The plots of ^*any*^EF and ^*all*^EF show the extent by which the ALM is increased by SL for a given experiment budget. During initial learning, these results are highly variable, and the reduction in variability coincides with consistent observation of larger than unity EF, suggesting the presence of a model-specific critical number of learning cycles to obtain a well-behaved, predictive ML model. This critical cycle number appears to be 10 cycles for RF and 30 to 40 cycles for GP and LE. For LE, the performance in the initial cycles is typically poor due to the use of pseudo-inversion methods until the training set consists of at-least one non-zero data point for each composition dimension, and in this case approximately 40 cycles seem necessary for any reasonable prediction. As a result, this ML model is best suited for applications with substantial experiment budget and where the computation time for model update and sequential prediction is at a premium. The GP model appears to have a similar minimum number of experiments before substantial model improvement, making RF methods a prudent choice for settings with low experiment budget.

The accelerate factor (AF) data provide a more direct measure of how SL can accelerate research. For example, consider scenarios where a research desired a (i) 50% or (ii) 90% probability of finding a top catalyst, or to find (iii) 50% or (iv) 90% of the top catalysts. For scenario (i) RF accelerates by a factor of 3 while GP and LE accelerate by a factor less than 2. For scenario (ii), RF performs similarly with a median AF of 2 while GP and LE accelerate by factors of 3 to 5. It is notable that for this most straightforward catalyst discovery task, acceleration by a factor of 10 is beyond the capabilities of these SL models. That level of acceleration is exhibited for scenarios (iii) and (iv) by many RF and GP-based learning runs, whereas LE exhibits more moderate ^*all*^AF of 4 to 6 for these scenarios. Collectively these results indicate that optimal choice of SL model varies with experiment budget and/or research goal, and that the maximum obtainable acceleration compared to random sampling is not very significant for some research tasks.

The qualitative similarity in performance of the three ML models motivates investigation of SL's sensitivity to both the acquisition function and the dataset. To benchmark this variability, we expand the simulated SL to four different explore-exploit hyperparameter values and to the four different datasets noted in Table 1. This set of 16 SL settings is analyzed using the random forest ML model due to its excellent performance and relatively fast execution, which facilitates continued use of 50 random initializations for each setting to characterize variability within a given setting. Using a single ML model also facilitates further quantitative analysis of the prediction quality, which we continue to measure using MAE of the test set. This MAE for different ML models is shown in Fig. 3 without comparison to random sample selection, a comparison that is not straightforward because the MAE values for random selection need to be simulated. For the RF model, the average MAE over 50 random experiment sequences was calculated for each of the 4 datasets to provide the random-selection baselines. To convert the MAE values from both SL and random selection into the metric ^*model*^AF, they are compared to the minimum expected MAE value for random selection, which is taken to be the average value for the 30% test set with 70% randomly selected train set. Like ^*any*^ALM and ^*all*^ALM, this ^*model*^ALM is between 0 and 1 for random selection, but unlike those other ALMs the value can exceed 1 during SL when sample selection provides a more predictive RF model for the test set compared to the RF model with random 30% test set.

**Fig. 5:**
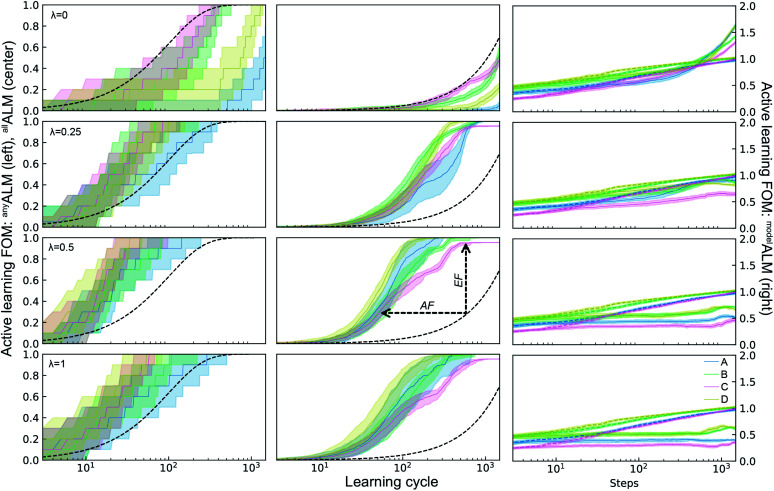
Active learning metrics (^*x*^ALM) over 1800 measurement cycles using a random forest ML model with different acquisition function hyperparameters *λ*. Each panel includes results for the 4 datasets describes in Table 1. The median value (solid line) as well as the 6^th^ to 94^th^ percentile (shaded region) are based on 50 random initializations of each sequential learning model for each dataset. The black dashed line shows the expectation value for random sampling (for ^*de*^ALM, ^*any*^ALM, ^*all*^ALM), and the colored dashed lines show the median value for 50 random initializations (for ^*model*^ALM).

**Fig. 6:**
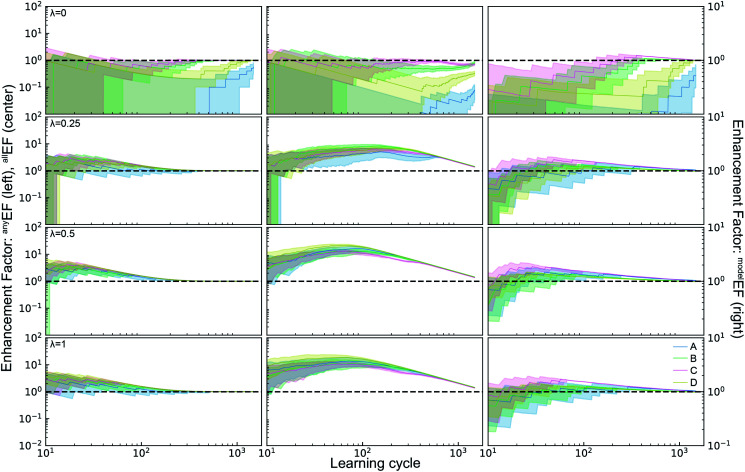
Enhancement factors for the active learning metrics ^*any*^ALM, ^*all*^ALM, and ^*model*^ALM for four different hyperparameters *λ* = 0, 0.25, 0.5, 1 of the acquisition function. Except for *λ* = 0, ^*any*^ALM and ^*all*^ALM are enhanced by factors of 1 − 30*x*. The explorative mode of *λ* = 0 is however the only mode in which ^*model*^ALM can reach enhancement factors above 1. The enhancement factor for ^*model*^ALM captures the fact that the *λ* = 0 produced a 50% better ^*model*^ALM than random selection of samples.

**Fig. 7:**
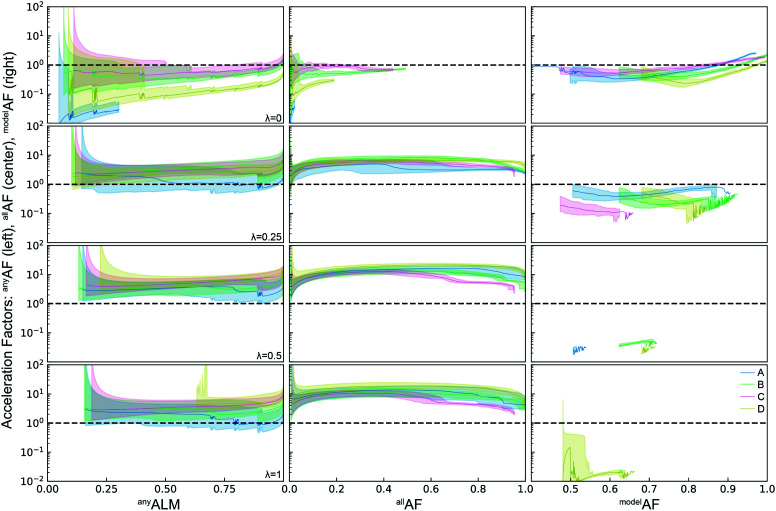
Acceleration factors for the active learning metrics ^*any*^ALM, ^*all*^ALM, and ^*model*^ALM for four different hyperparameters *λ* = 0, 0.25, 0.5, 1 of the acquisition function. Except for *λ* = 0, ^*any*^ALM and ^*all*^ALM are accelerated by factors of 1 − 30*x*. The explorative mode of *λ* = 0 is however the only mode in which ^*model*^ALM can reach values above 1 (not shown). The equal weighted and exploitative mode of *λ* = 0.5, 1 fail to select samples to improve the models and result in overall low ^*model*^ALM.

The ALMs, EFs, and AFs for the 16 settings are shown in Fig. 5, 6 and 7, respectively, where in each figure the 3 columns correspond to *any*, *all*, and *model* metrics; the 4 rows correspond to the *λ* hyperparameter values; and the 4 catalyst datasets are distinguished by color in each panel. The enhanced discovery of any or all top catalysts is qualitatively similar, with EF and AF values consistently larger for the more challenging task of discovering all top catalysts. Partial to full exploitative sample selection can accelerate the discovery of any top catalyst by factors of 1 to 10 depending on dataset and random initialization. This variability is smaller for identifying all top catalysts where improvements by factors of 3 to 10 are routinely observed in both the fraction of top catalysts discovered and the number of cycles required to discover them. Importantly, these enhancements are most pronounced for experiment budgets near 100 where SL has consistently identified at least half of the top catalyst, and there are diminishing returns of sequential learning as the number of experiments grows larger. Choosing (*λ* = 0.5) in the acquisition function provides a nice balance of improvement with relatively little variability with respect to dataset and random initialization, making this a suitable choice of hyperparameter for these types of catalyst discovery tasks.

Due to poor model accuracy during initial cycles, all hyperparameters have a flavor of random selection at the onset, motivating commencement of plotting at 10 cycles in Fig. 6. For *λ* = 1, this initial random-like selection likely facilitates model learning of the dynamic range of the catalytic activity, indicating that models that become predictive more quickly could performance worse against some metrics. The only substantially different hyperparameter is the uncertainty based exploration (*λ* = 0), which is intuitive given that selecting catalysts based on model uncertainty isn't optimal for identifying top catalysts, and in fact this mode of SL decelerates discovery of top catalysts. For the task of learning a predictive model, as measured by ^*model*^ALM, this explorative sequential learning performs somewhat better than, but often similar to, random sample selection. The values of ^*model*^EF and ^*model*^AF are consistently above 1 (better than random) for learning cycles above 300 to over 1000 depending on the dataset, or target relative MAE (^*model*^ALM value) above 0.86 to 0.97 depending on the dataset, respectively. That is, SL only outperforms random selection for model quality when the experiment budget is a substantial fraction of the search space. The situation is far worse for the larger hyperparameter values corresponding to partial to full exploitation, where sequential learning underperforms random selection under any experiment budget or desired MAE value. These results are emblematic of the common knowledge that uniform sampling is a reasonable strategy for predictive model building, and that bias based on performance is detrimental to model building.^35,36^

## Discussion

The compendium of simulated learning results indicate that (i) exploration by uncertainty-based sample selection can accelerate the establishment of predictive models in niche situations where a substantial fraction of the search space is measured, however random experiment selection is typically a suitable strategy; (ii) EFs and AFs up to approximately 20× are possible for identifying any or all top catalysts, demonstrating a ceiling for the extent by which sequential learning can improve catalyst discovery; (iii) EF and AF values well below 0.05 are also observed, indicating that the floor for deleterious effects of sequential learning is relatively deep compared to the ceiling. That is, poor choices for ML model and/or acquisition function for a given experiment budget or research object can lead to substantially worse performance than random sample selection, a critical lesson that illustrates the importance of comprehensive workflow design in the context of specific research objectives.^2^

We also note that these accelerations are with respect to random sample selection, which is not a commonly applied experiment design strategy. Development of more illustrative baselines is an important area for future research, although we note that comparing to traditional human-rational catalyst selection will also motivate further development of the acceleration metrics, as the relative costs of the experiment design mechanisms should be considered. In the present work, the SL algorithms and random selection are both automated without human intervention, making them equivalent in terms of human effort per sample selection, and the additional computational expense of SL compared to random selection is not considered in the benchmarking metrics.

The design of an appropriate SL algorithm must be performed in the context of the research task at hand, which is consistent with general best practices in design of experiments. The *any*, *all* and *model* active learning metrics of the present work are designed to span a range of common research goals from the most applied to the more fundamental. If a material is needed to enable a technology, one could employ a search to find any single such material, which for the present data is emulated by a search for any of the catalysts in the top percentile of activity and calculated as the probability of finding a top catalyst by a given SL cycle. A more general materials discovery effort would aim to identify all good materials, which for the present data is quantified by the fraction of the top percentile of catalysts identified by a given SL cycle. A more general materials exploration study would aim to predict the performance of all materials in the search space, as quantified by the model MAE. For catalyst science, or more generally for basic research, the ability to predict all catalyst activities would provide composition–property relationships that characterize the underlying chemistry. Ultimately, the desired outcome is a fundamental description of the mechanisms underlying compositional variation in catalytic activity. Benchmarking SL in this context is not addressed in the present work due to the lack of a objective metric that is aligned with this research goal. The ability of SL to accelerate knowledge discovery remains an outstanding question with important implications for its applicability in accelerating fundamental research.

Additional considerations in the choice of SL model that are not addressed in the present work due to their specificity to a given application are (i) the time available for SL calculations in the experimental loop and (ii) either incorporating known, or predicting on-the-fly, the uncertainty of individual measurements, including outlier detection. The ML models in order of computational expense are LE, RF and GP, although calculation times will vary with specific implementations of these models, prompting our focus on the performance aspects related to accelerated discovery.

While the benchmark data in the present work involves discovery of heterogeneous electrocatalysts, the ML models and SL acquisition functions are agnostic to their application for materials discovery. Construction of materials and catalysis-aware search spaces, ML models, and sample selection policies will be necessary to accelerate research by more than a factor of 10 for the type of research discussed in the present work. Our use of datasets covering a multitude of composition spaces is intended to make these observations generally applicable to materials discovery. Adding axes with highly nonlinear behaviour to the search space such as processing or device-related parameters is likely to make random sample selection less effective, creating the opportunity for SL to be drastically more impactful. For example, recent reports of SL for the synthesis and casting of organic thin films^20^ has shown enhancement factors in excess of 30×^37^ compared to a comprehensive (conservatively chosen) grid search sampling. As the community continues to establish benchmarks for evaluating SL techniques, it is important to consider the amount and type of data that is required to establish accurate benchmarks. For example, using a search grid that oversamples the search space with respect to the scale at which FOM variations occur can make comprehensive experimentation appear arbitrarily bad compared to SL or even random selection, *i.e.* make SL appear artificially effective compared to comprehensive grid search. We note that the benchmarking of the present work capitalizes on the knowledge gained through millions of experiments over the past 7 years including sampling at finer composition intervals,^32,33^ which revealed that the 10 at% steps of the present work to be a suitable grid for this type of catalysis research. Without these types of comprehensive high throughput experiments, or at least experimentation well beyond that of a given SL method, the efficacy of the sequential learning method cannot be appropriately benchmarked. These observations demonstrate the complementary roles of high throughput experimentation and SL, motivating a research strategy that incorporates both methods to develop optimal discovery strategies and to identify outstanding challenges in AI-guided experimentation.^3^ Such challenges will motivate algorithm development to substantially accelerate research and realize the paradigm shift in materials discovery that is envisioned by early adopters of ML for materials science.

Initial areas for algorithm development identified by the present work include improvements to accurate quantification of uncertainty and expansion of the purview of SL. Exploitation-based approaches are likely to perform reasonably well in simple search spaces. However, their efficacy is likely to be lower in complex search spaces wherein the role of exploration and accurate uncertainty quantification becomes essential. In this work, exploitation-based sample selection was not found to be optimal in any setting, highlighting the opportunity for improving performance of exploration and expected-improvement approaches *via* better uncertainty quantification. The computationally inexpensive methods such as LE and RF use standard deviation over a collection of estimators to quantify uncertainty, and given the general overconfidence of these methods (under-estimation of uncertainty),^38^ uncertainty calibration or other methods for improving uncertainty quantification are expected to be quite impactful. Random forests have shown to outperform LE and GP for small datasets, necessitating further research on the role of ML models that include bagging and boosting in accelerating materials research for low-throughput and/or small experimental budget settings. As discussed in a recent critical review of the use of automation and active learning in materials science experimental workflows,^2^ the importance of SL of a given task must be evaluated within the context of the larger workflow containing that task. In the present example, batch synthesis of the composition grid within the given composition system and parallel processing of the catalysts means that the synthesis portion of the workflow is not accelerated by the catalyst-sequential learning. The acceleration factors of the benchmarking in the present work apply onto to the serial electrochemistry that provides the catalyst activity, meaning that the acceleration factors for the entire experiment workflow will be even smaller than the ceiling of approximately 20-fold acceleration observed in Fig. 4 and 7. For workflows that combine parallel synthesis experiments and serial experimentation, comprehensive ML strategies that can suggest new synthesis experiments (*e.g.* composition spaces and/or processing conditions) need to be developed and combined with the SL strategies for serial experimentation. Substantial advancement in ML algorithms and the design of the search space are required to realize this more global strategy for AI-guided experiments.

## Conclusions

Benchmarking of sequential learning methods for catalyst discovery can accelerate research, but not yet at the orders-of-magnitude level anticipated for AI-guided discovery. The performance of sequential learning algorithms depend varies more with respect to the research goal than the specifics of the model, as demonstrated by exploring three complementary research goals (discover any good catalyst, discover all good catalysts, discover a predictive model) and three complementary ML models (random forest, Gaussian process, and linear ensemble). The variability in performance of models for four catalyst datasets with different composition spaces reveals consistent qualitative trends, indicating some level of generality for the observations. The task of finding any good catalyst is difficult to accelerate with sequential learning, as 10 to 100 samples are required for training robust models, and random selection has an appreciable chance of discovery within that many cycles. Finding all good catalysts is more challenging and is where sequential learning can most substantially outperform random selection, producing up to 20 times acceleration for specific settings. Finding a predictive model, which is often aligned with basic research that seeks to understand the activity of all catalysts, is generally not accelerated by sequential learning, motivating algorithm development in this area with particular focus on uncertainty quantification. While the search space of the benchmarking data is of appreciable size with 2121 unique catalysts per dataset, and of appreciable dimensional depth with 6 metal cations in the metal oxide composition space of each dataset, the search space of all possible catalysts with additional axes for processing parameters and electrode preparation is much larger. AI-guided discovery may offer greater levels of acceleration in these larger search spaces, motivating establishment of compact representations that facilitate model training with sparse data and enable prediction into composition axes with little to no training data.

## Data and code availability

All catalyst data is visualized in Fig. 2 or the ESI† and is available for interactive visualization and download at http://data.matr.io/ACE-I. Source code for benchmarking sequential learning runs against random sample selection and demonstrating the sequential learning is available at https://github.com/SantoshSuram-TRI/ACE-I. The compilation of data is available in that repository and also at https://data.caltech.edu/records/1345 (DOI: 10.22002/D1.1345).

## Conflicts of interest

B. R., H. S., S. S. and J. G. filed a provisional patent application on active learning enabled experimental catalyst materials discovery: US app. no. 62/837,379. The remaining authors declare no competing interests.

## Supplementary Material

SC-011-C9SC05999G-s001
