# Supplementary material for: Benchmarking the acceleration of materials discovery by sequential learning
Source: Chem Sci. 2020 Jan 29;11(10):2696–706. doi: 10.1039/c9sc05999g (PMC8157525; doi:10.1039/c9sc05999g)
Supplement: SC-011-C9SC05999G-s001 [file SC-011-C9SC05999G-s001.pdf]

Electronic Supplementary Material (ESI) for Chemical Science.
This journal is © The Royal Society of Chemistry 2020

# 1 Supplementary Information: Benchmarking the acceleration of materials discovery by sequential learning

## Averaging Procedure

To created more continuous evaluation of the inherently discrete process of identifying top catalysts, we deployed the following binning strategy to characterize the median ALM as well as its variability using the 50 random initializations per setting (ML model, $\lambda$ values, and dataset). For binned averaging, the 50 sequential learning runs were split into 5 different bins such that all runs belong to only one bin. For each bin the average of the active learning metric was taken, which results for ${}^{any}ALM$ in a increment of 0.1 instead of the binary 0/1 of having found a top percentile sample. For the chosen split into 5 bins this results in a total of five ${}^{x,ave,j}ALM$. This procedure is performed 100 times with random bin assignment to yield a total of 500 values of ${}^{x}ALM$ from which the median and percentile bands are calculated. The median and percentile bands of the enhancement and acceleration factors are calculated from comparing median and percentile bands of the ALM to the random baseline.

## Expectation Value Baselines

For ${}^{any}ALM$ and ${}^{all}ALM$, the expected values as a function of learning cycle can be derived via probability analysis. Let $N$ be the size of the search space and $M$ the size of the exclusive class, in this case $M = 0.01 \times N \approx 21$ for the top percentile of catalysts. Then the probability analysis is as follows:

1. $P_i$ if the probability that cycle $i$ selects from the exclusive class
2. $E_i$ is expected number from exclusive class sample that have been selected by cycle $i$
3. $A_i$ is the probability that cycle $i$ is the first where a sample from the exclusive class is selected
4. $cA_i$ is the cumulative probability of $A_i$, which is the probability that any from exclusive class have been measured by cycle $i$.
5. For $i = 0$, $P_i = E_i = A_i = M/N$
6. For subsequent cycles, iterative updates are as follows:
   (a) $P_i = \frac{M-E_{i-1}}{N-i}$
   (b) $E_i = \sum_{n=0}^{i} P_n$
   (c) $A_i = P_i \times \prod_{n=0}^{i-1}(1-P_n)$
   (d) $cA_i = \sum_{n=0}^{i} A_n$
7. The expectation value of ${}^{any}ALM$ is $E[{}^{any}ALM_i] = cA_i$.
8. The expectation value of ${}^{all}ALM$ is $E[{}^{all}ALM_i] = E_i/M$.

Fig. S1 includes comparison of simulated random selection and the corresponding expectation value baselines, showing agreement within the resolution of the simulation.

## Comparison of Expectation Value Baselines and averaging

As an empirical justification the figure below shows the averaging results for random sample selection by plate in the same color scheme as in the main text as dashed lines. There is virtually any difference between the random sample selection and rhe random expectation value baseline justifying the averaging procedure.

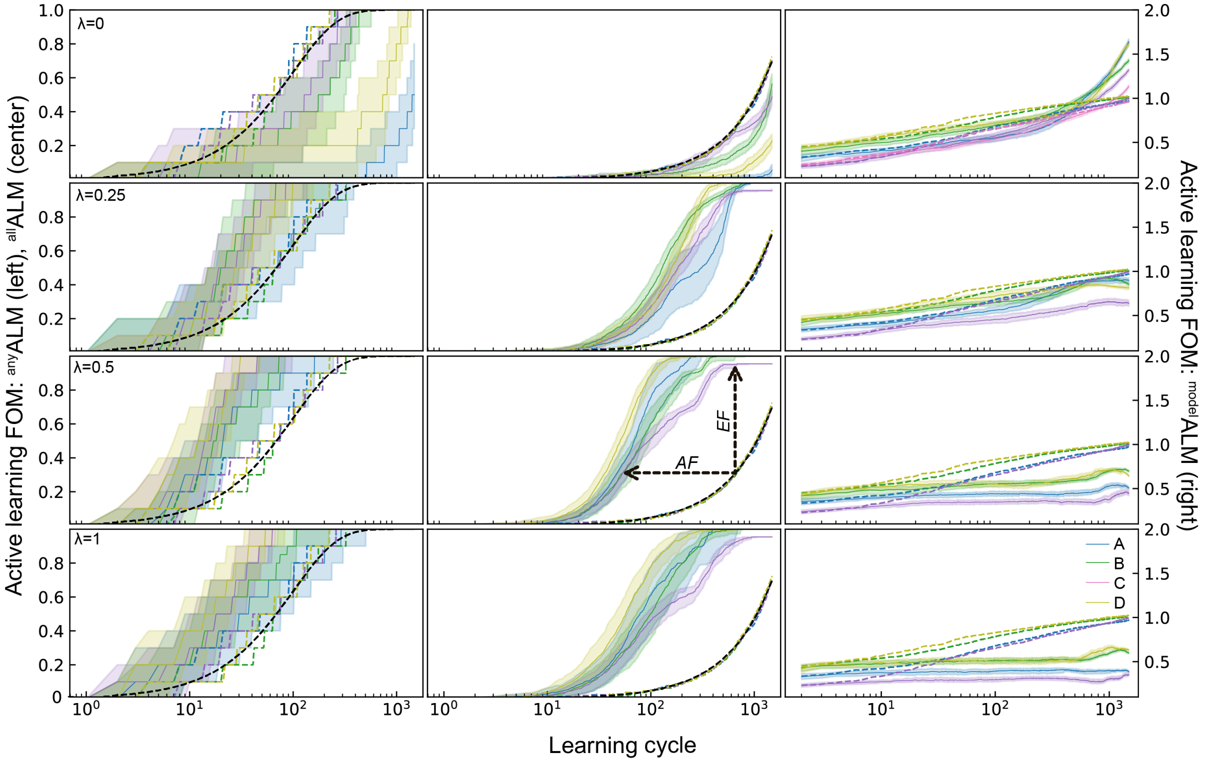


**Fig. S1** Active learning metrics vs. learning cycle, similar to the main paper but with the additional dashed,colored lines for simulation of random sampling for each plate, which generally follow the closed form expression but with discrete jumps in the ALM values, as described in the main text.

**Catalyst activity plots**

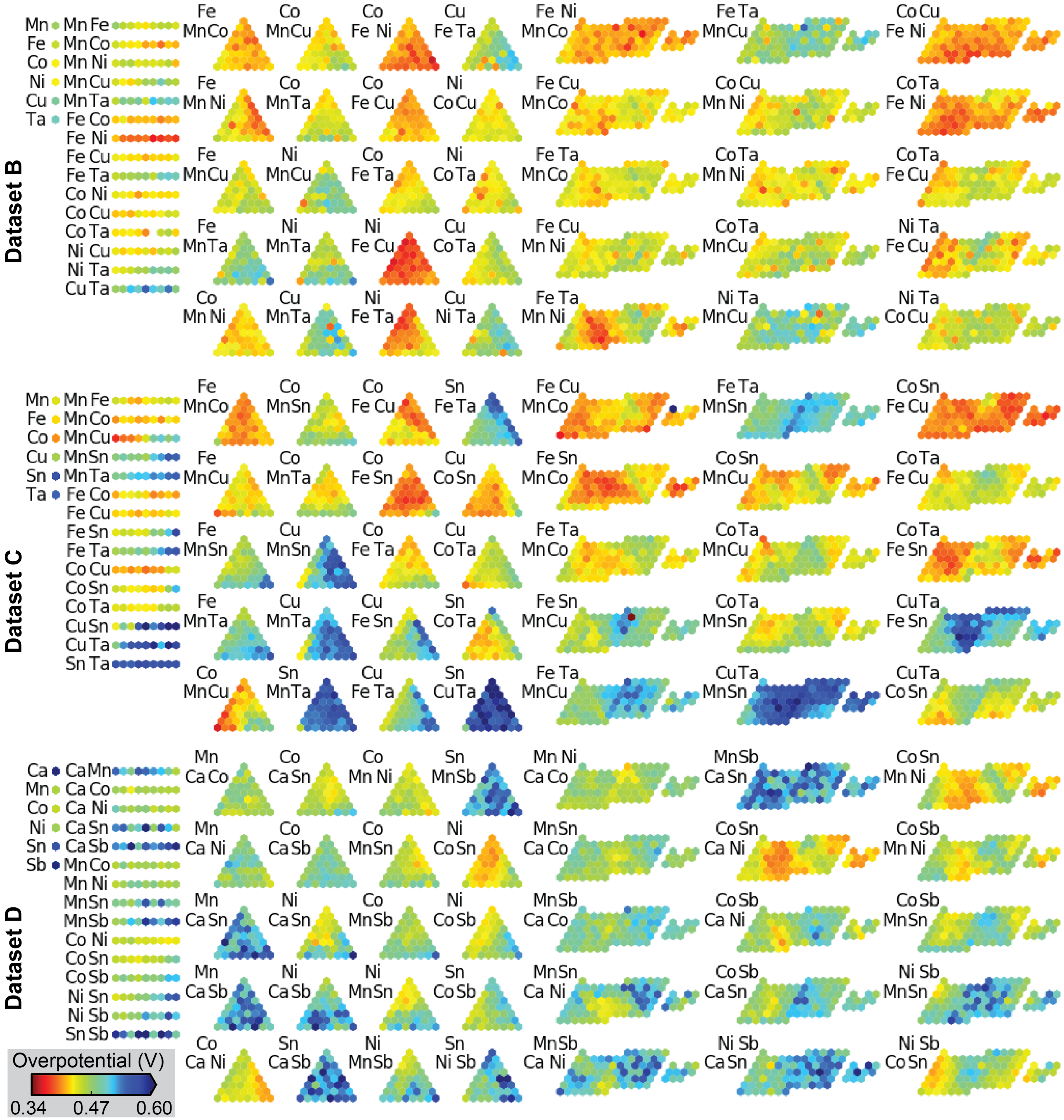


**Fig. S2** The catalyst FOM (overpotential $\eta$ to reach $3mA/cm^2$) is shown using the composition plotting scheme illustrated with dataset A in the main paper. The common color scale appears at bottom left.
